# Re-exploring the core genes and modules in the human frontal cortex during chronological aging: insights from network-based analysis of transcriptomic studies

**DOI:** 10.18632/aging.101589

**Published:** 2018-10-20

**Authors:** Mulin Xu, Yu Liu, Yi Huang, Jinli Wang, Jinhua Yan, Le Zhang, Cuntai Zhang

**Affiliations:** 1 Department of Geriatrics, Tongji Hospital of Tongji Medical College, Huazhong University of Science and Technology, Wuhan 430030, P.R. China; 2 Department of Internal Medicine, University of Utah, Salt Lake City, Utah 84112, USA; * Equal contribution

**Keywords:** frontal cortex aging, network-based analysis, HDAC1, CDC42, YES1

## Abstract

Frontal cortical dysfunction is a fundamental pathology contributing to age-associated behavioral and cognitive deficits that predispose older adults to neurodegenerative diseases. It is established that aging increases the risk of frontal cortical dysfunction; however, the underlying molecular mechanism remains elusive. Here, we used an integrative meta-analysis to combine five frontal cortex microarray studies with a combined sample population of 161 younger and 155 older individuals. A network-based analysis was used to describe an outline of human frontal cortical aging to identify core genes whose expression changes with age and to reveal the interrelationships among these genes. We found that *histone deacetylase 1* (*HDAC1*) and *YES proto-oncogene 1* (*YES1*) are the two most upregulated genes, while *cell division cycle 42* (*CDC42*) is the central regulatory gene decreased in the aged human frontal cortex. Quantitative PCR assays revealed corresponding changes in frontal cortical Hdac1, Yes1 and Cdc42 mRNA levels in an established aging mouse model. Moreover, analysis of the GSE48350 dataset confirmed similar changes in HDAC1, CDC42 and YES1 expression in Alzheimer's disease, thereby providing a molecular connection between aging and Alzheimer's disease (AD). This framework of network-based analysis could provide novel strategies for detecting and monitoring aging in the brain.

## Introduction

Human cognition relies to a great extent on the function of the frontal cortical lobe, which is disproportionately enlarged in humans compared to other primates. Moreover, age-associated cognitive deficits, including forgetfulness, distractibility, inflexibility, and impaired executive functions, all likely reflect frontal cortical dysfunction [1–7]. This reduced cognitive function in older people predisposes them to neurodegenerative diseases, such as Alzheimer's disease (AD) and Parkinson's disease. Understanding the genetic causes behind aging could potentially provide an effective way to detect and monitor the progression of aging, which could enable people to grow old more gracefully.

Despite the significance of brain aging, the existing knowledge on the molecular mechanisms underlying biological aging remains limited, especially in humans. This is in part because most studies basically entail a comparison between “young” and “old” individuals, and it is a challenge to obtain samples from older but healthy adults. Another important difficulty is the heterogeneity among studies. This inconsistency is most likely due to variation in the techniques used, limited study size, low signal-to-noise ratios, and differences in the subpopulations typically observed when analyzing the aging transcriptome. Consequently, an integrated analysis of the aging transcriptome is a promising alternative to classical individual gene analyses. Such integrative methods for analysis of big data have been successfully applied to disease subtyping, biomarker discovery, and drug repurposing [8–10]. Effective and objective tools for combining big data are the key to future success in health informatics.

To comprehensively evaluate the relevant public data concerning age-associated gene expression in the human brain, an effective method for integrating information mined from multiple datasets is needed. Recently, a network approach constructed using protein-protein interaction (PPI) data has been developed to interpret the interactive patterns within large datasets. This PPI network analysis was successfully applied for detection of new and hub changes in the human transcriptome [11,12]. This approach shows great promise as a robust method for integrating gene expression data and providing insight into complex human diseases. Applied to integrating age-associated data, this method exhibits high reproducibility across multiple expression datasets [13]. The results obtained so far provide a molecular basis for future research into the mechanisms underlying human aging, which could potentially guide individual anti-aging treatment decisions [13]. Importantly, however, although screening big data can yield a list of candidate biomarkers, determining the functional role of each gene will require further validation in animal models and clinical samples.

To characterize the aging-associated changes in the frontal cortex, we first built a compendium of genes related to chronological age using gene expression profiles from five microarray studies [14–18]. We then used PPI network-based meta-analysis to detect the core genes and functional modules. All the candidate genes were further confirmed using an established mouse model of aging and qPCR methods, which showed similar changes in the frontal cortex of this mouse model. Cognitive function and spatial learning were verified using the Morris water maze test (MWM). Finally, we confirmed these results by screening the GenAge database and analyzing changes in the expression of these genes in AD samples.

## RESULTS

### Overlap of differentially expressed genes among studies within the compendium

**Figure 1:**
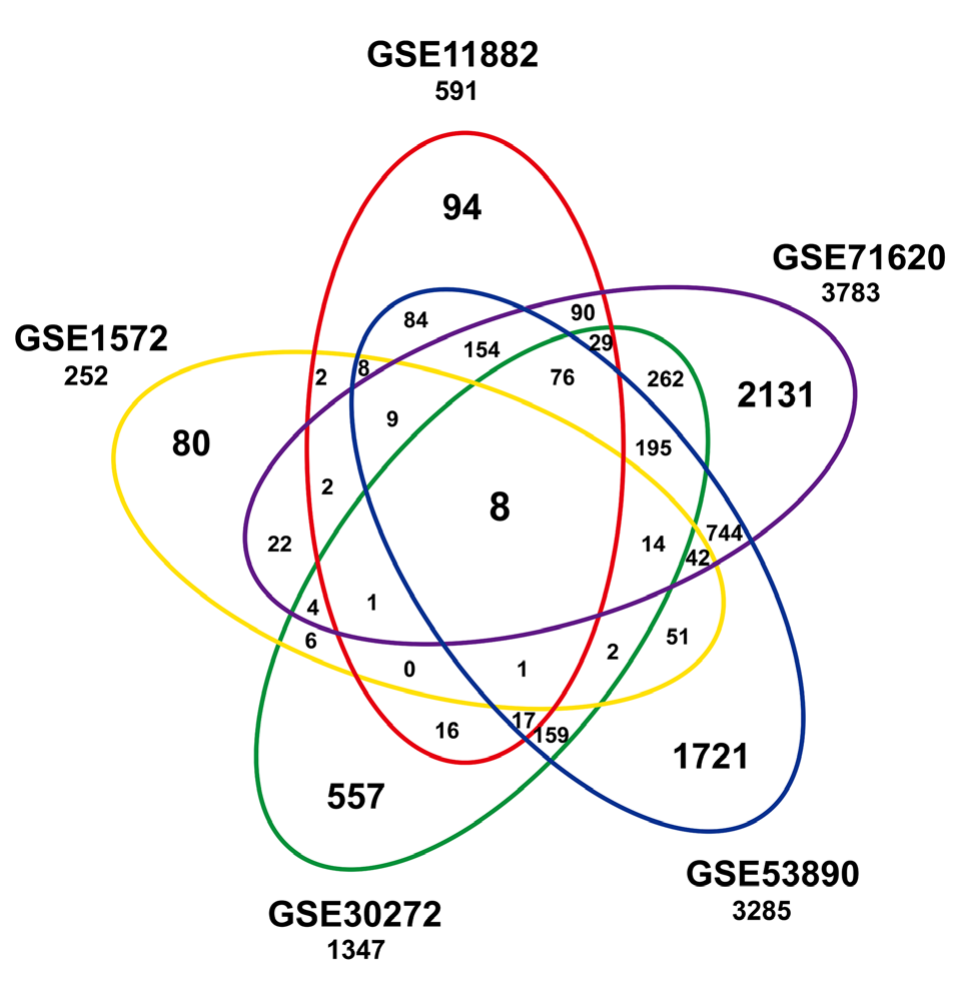
**Significant age-associated genes in studies of the frontal cortex compendium.** Shown is a Venn analysis performed to determine the overlap of significantly age-associated genes identified in five independent studies.

To identify a common transcriptional signature reflecting age within the frontal cortex, we built a gene expression compendium using five independent studies (Table 1). Data were extracted and annotated, yielding a compilation of 7274 unique genes from 316 individuals, including 161 younger and 155 older individuals. An integrative analysis across datasets was then performed by computing the differentially expressed genes per dataset and assessing the overlap of the significant results. Eight genes (*HTR7P1, MMD, OLFM1, ATP2B2, ANXA4, LPL, VCAN,* and *RHOBTB3*) were significantly associated with age among the five datasets (Figure 1 and Table S1). Among them, *LPL* (*lipoprotein lipase*) is mainly expressed in adipose tissue and regulates the availability of polyunsaturated fatty acid within the central nervous system. *HTR7P1*, *OLFM1* and *ATP2B2* are highly expressed specifically in brain.

**Table 1:** Characteristics of studies composing the prefrontal cortex gene expression compendium.

**Study(Citation)**	**Dataset**	**Platform**	**Region**	**Types of sample**	**Sample (young20-40)**	**Sample (old60-90)**
Lu T, 2004	GSE1572	Affymetrix Human Genome U95 Version 2 Array	Boston, MA USA	human frontal cortex tissue	9	12
Berchtold NC, 2008	GSE11882	Affymetrix Human Genome U133 Plus 2.0 Array	Irvine, CA USA	Human superior frontal gyrus brain tissue	13	18
Colantuoni C, 2011	GSE30272	Illumina Human 49K Oligo array (HEEBO-7 set)	Baltimore, MD USA	human prefrontal cortex tissue	52	22
Lu T, 2014	GSE53890	Affymetrix Human Genome U133 Plus 2.0 Array	Boston, MA USA	human frontal cortex tissue	13	13
Chen CY, 2016	GSE71620	Affymetrix Human Gene 1.1 ST Array [transcript (gene) version]	Pittsburgh, PA USA	human prefrontal cortex tissue	74	90

### Meta-analysis of the frontal cortex throughout the normal aging

**Figure 2:**
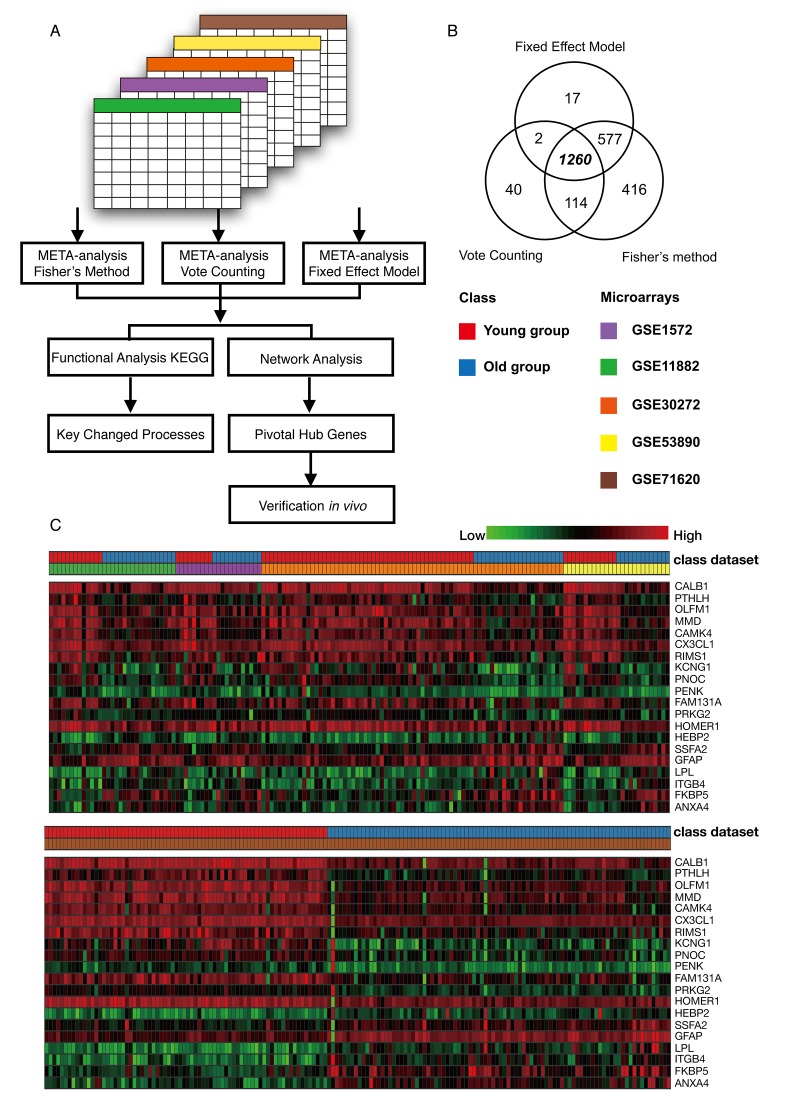
**Meta-analysis of the frontal cortex throughout normal aging.** (**A**) Flowchart of the meta-analysis. (**B**) Venn diagram of differentially expressed genes identified from the meta-analysis using Fisher’s method, the vote counting method and a fixed effect model. (**C**) Heat map representation of the top 20 differentially expressed genes across different microarrays identified from the meta-analysis (row-wise comparison).

Five microarrays were analyzed using Integrative Meta-Analysis of Expression Data (INMEX), a web interface for integrative meta-analysis. The overall meta-analysis workflow in this study is shown in Figure 2A. By employing three meta-analysis methods, Fisher's method, Fixed effect model and Voting count, we identified 2367, 1856 and 1416 differentially expressed genes, respectively (Combined p < 0.001 or vote counts ≥ 2 were considered to be significant). Among those, 1260 genes were identified by all three methods (Figure 2B). A Venn diagram of these data was shown in Figure 2B. Among this group, 635 (50.4%) genes were downregulated and 625 (49.6%) were upregulated in older group as compared to the younger group. A heat map visualization of the top 20 genes across the different studies is displayed in Figure 2C. Among them, *ANXA4* (*annexin A4*) was the top upregulated gene, and *CALB1* (*calbindin1*) was the most significantly downregulated gene across the five microarray datasets. A complete list of the differentially expressed genes is provided in Table S2. The merged data of this meta-analysis is listed in Data S1.

### Functional enrichment of differentially expressed genes analysis

**Figure 3:**
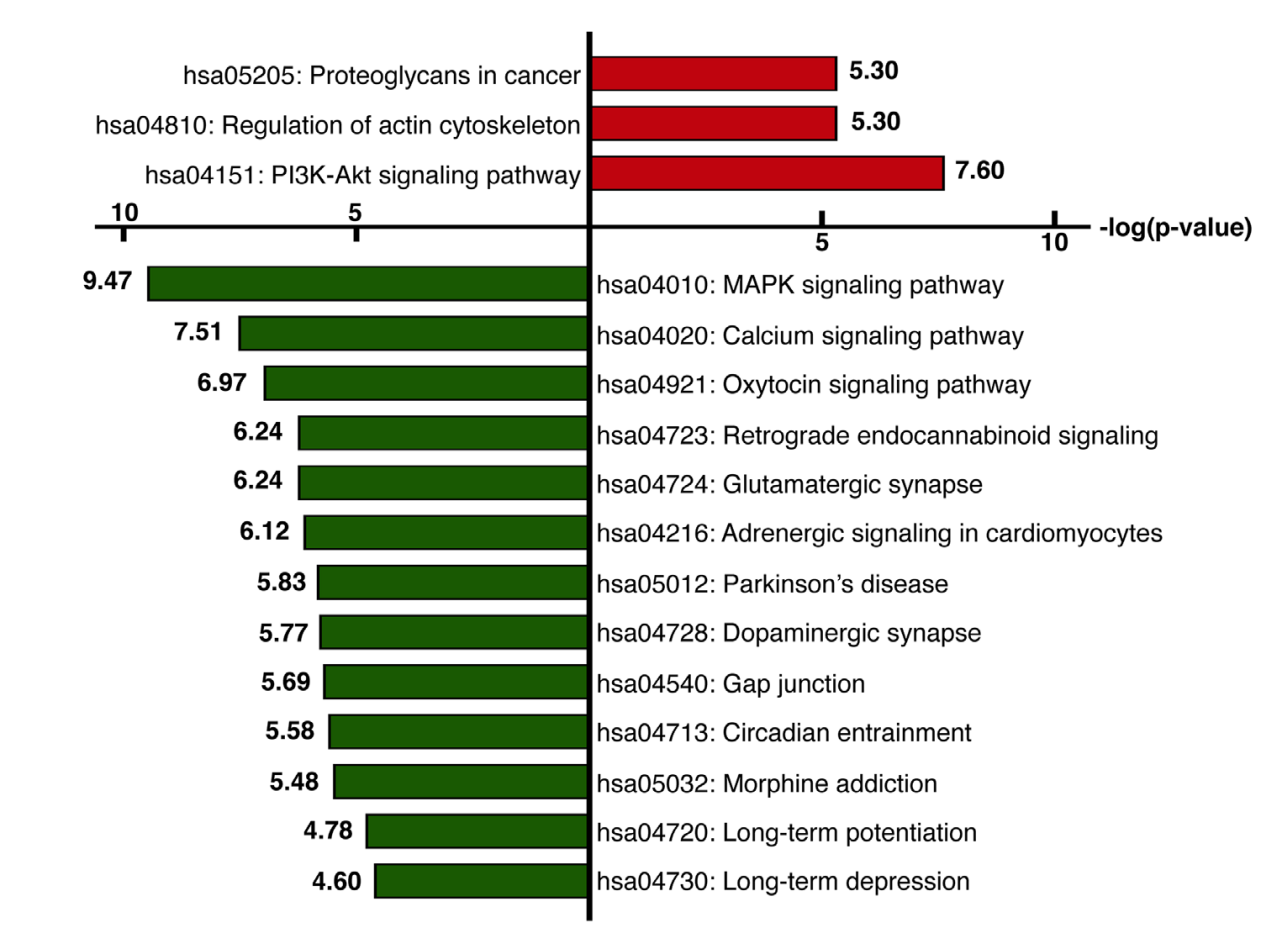
**KEGG pathway analysis.** KEGG pathway analysis was performed using the set of genes showing upregulated (red) or downregulated (green) expression (FDR< 0.05). The x-axis represents the fold enrichment (indicating the magnitude of enrichment in our dataset against the population background based on analysis using DAVID bioinformatics tools). The y-axis shows the pathway categories.

**Figure 4:**
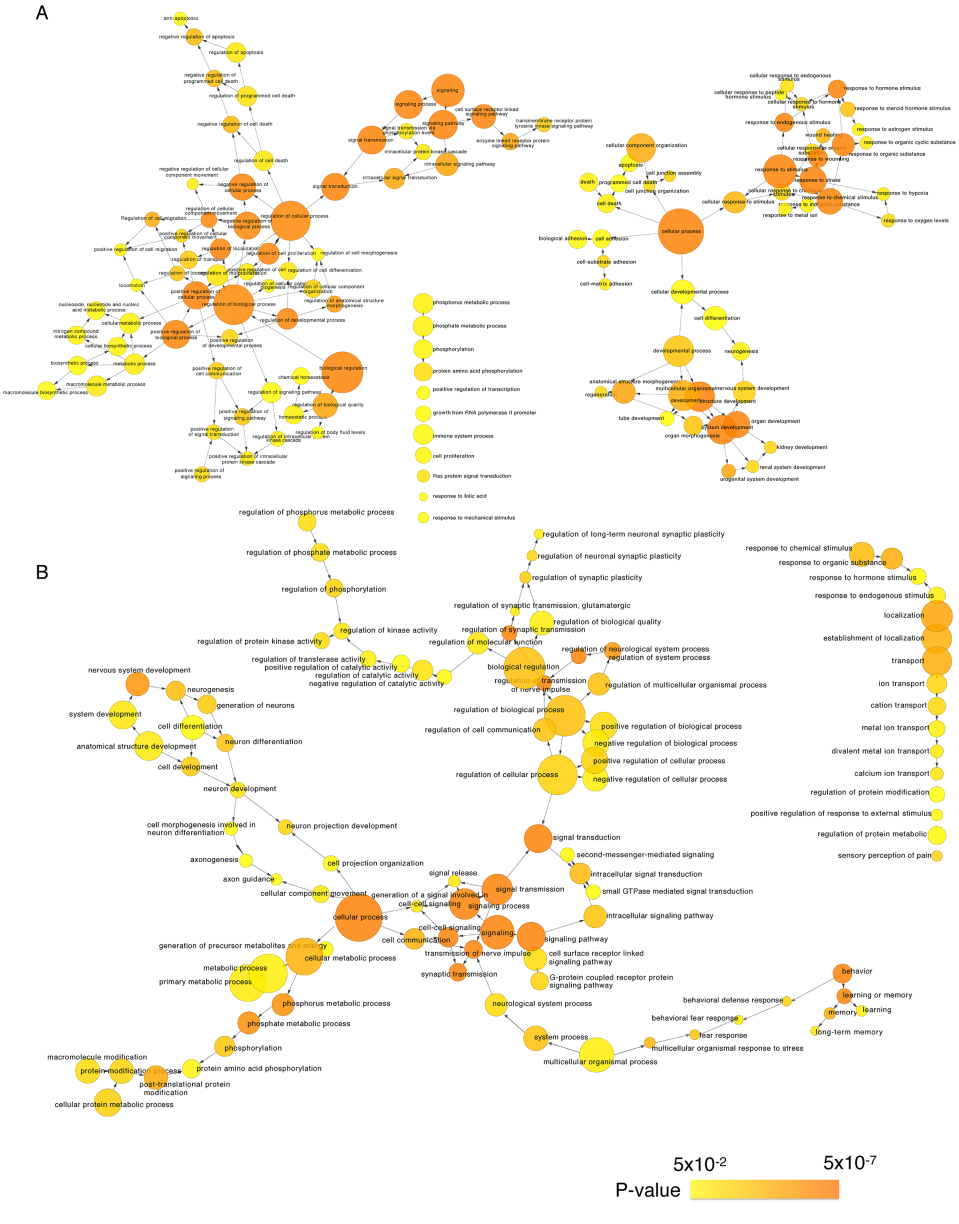
**Gene Ontology analysis of significantly age-associated genes in the frontal cortex.** Shown is a BiNGO (the Biological Network Gene Ontology tool) analysis depicting upregulated (**A**) and downregulated (**B**) genes in over-represented categories in the ontology of biological processes. The size of the nodes is proportional to the number of genes in the test set that are annotated to that node. Colored nodes are significantly over-represented along a color scale ranging from yellow (p = 0.05) to dark orange (p = 5.00E-7).

We next identified the enriched functional groupings that were significantly associated with age in the human frontal cortex. Kyoto Encyclopedia of Genes and Genomes (KEGG) pathway analysis was performed using the set of up- and downregulated genes (False Discovery Rate (FDR) < 0.05) (Figure 3, Table S3). Among the enriched pathways were a number involving protein kinase signaling, including “PI3K-Akt signaling pathway” and “MAPK signaling pathway”. Enriched pathways were also related to chemical synapses (“Retrograde endocannabinoid signaling,” “Glutamatergic synapse” and “Dopaminergic synapse”) and electrical synapses (“Gap junction”). More specifically, MAPK and calcium signaling were shown to be involved in human longevity in an independent genome-wide association study (GWAS) of Han Chinese [19]. On the other hand, signal transduction systems that mediate “Long-term potentiation (LTP),” “Long-term depression (LTD)” and “Calcium signaling pathways” showed age-related downregulation. Using BiNGO in Cytoscape, we were able to obtain a global perspective of the changes in gene expression patterns (Figure 4). The gene sets showing upregulated expression were enriched for biological processes and molecular functions associated with “response to stimulus,” “cell migration,” “programmed cell death” and “apoptosis.” By contrast, the gene sets showing downregulated expression were enriched for biological processes and molecular functions associated with “synaptic transmission,” “phosphate metabolic process” and “learning and memory.” Given the functional enrichment of these differentially expressed genes, we delved further into the results in an integrative meta-analysis.

### Analysis of PPI networks of selected genes

**Figure 5:**
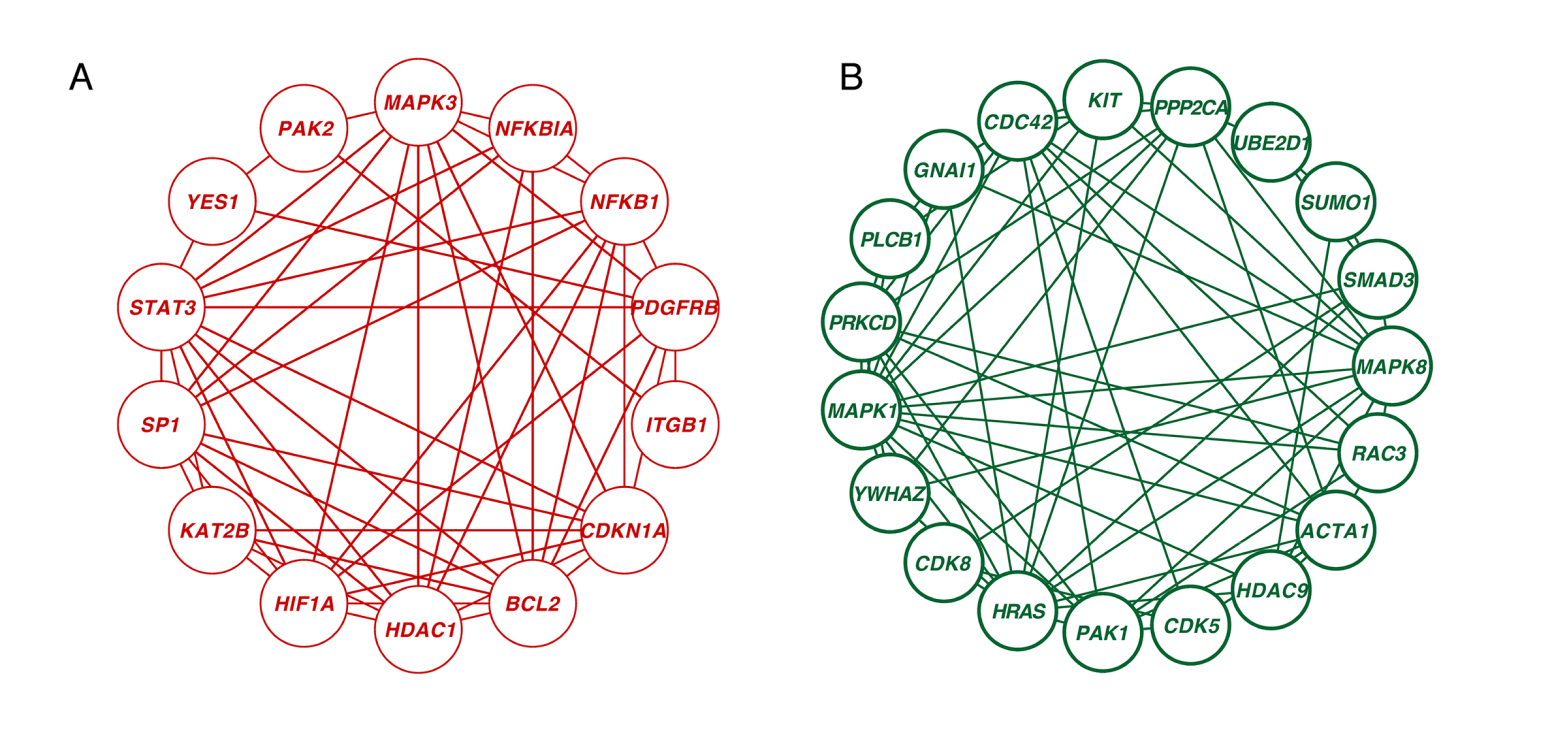
**Network analysis of significantly age-associated genes in the frontal cortex.** (**A**) Zero-order interaction network of upregulated genes (red). (**B**) Zero-order interaction network of downregulated genes (green).

A novel integrative PPI network-based approach was applied to explore all the corresponding genes found in our meta-analysis search. This method has been validated as a way of identifying age-associated bio- data [13]. Considering the betweenness (BC) and degree of centrality (DC), we ranked the 1260 genes that showed significant differences between young and old in our integrative meta-analysis. We screened 33 hub genes that had at least 10 node degrees and ranked the top 10 percent of the total genes. Among them, 14 genes were upregulated and 19 genes were downregulated in the older group as compared to the younger group (Figure 5, Table S4). *CDC42* was the most significantly downregulated hub gene (BC = 12193; DC = 30), followed by *MAPK1* (BC = 9241.11; DC = 29) and *HRAS* (BC =7136.24; DC = 27). *HDAC1* was the centermost upregulated gene (BC = 6305.53; DC = 25) followed by *YES1* (BC = 6574.41; DC = 22).

Neural aging is regulated through extensive interaction among genetic networks, signaling pathways and cellular metabolic responses, not through any single gene. However age-related genes are not spread throughout the interactome; instead, they cluster into tightly connected modules [20]. The joint expression of modules plays critical roles in human aging. After mapping the 1260 significant genes onto the PPI network, the zero-order network contained five subnetworks, including one big subnetwork (“continent”) (nodes 373, edges 743) and four smaller ones (“islands”) (p < 0.05, gene numbers ≥ 5). The big subnetwork was divided into 18 significant modules (p < 0.05, gene numbers ≥ 5) (Figure S1). In the following steps, we applied Gene Ontology (GO) analysis to these subnetworks and modules. The enriched terms characterizing the four satisfactory PPI islands and eighteen significant modules are shown in Table S5. The genes in module 1 were enriched for “apoptotic process.” Those in modules 2 and 3 were enriched for “regulation of transcription from RNA polymerase II promoter” and “regulation of small GTPase mediated signal transduction,” respectively. These enriched terms also included “semaphorin-plexin signaling pathway,” which is brain-specific expression and controls presynaptic neurotransmitter release and homeostatic plasticity [21]. In addition, three subnetworks were related to the mitochondria. Mitochondrial dysfunction is a critical characteristic of the aged brain and neurodegenerative diseases. This includes reduced respiration, dynamic structural modifications, loss of membrane potential and the changes of proteomic profile [22]. Structurally or functionally damaged mitochondria are more proficient at producing reactive oxygen species (ROS) but less efficient at ATP production, which accelerates pathological processes such as AD [23]. The remaining subnetwork was enriched for “mRNA splicing via spliceosome.” Of all the human organs, the brain has the largest amount of alternative splicing [24], and the genes that are alternatively spliced have been associated with neurodegenerative disorders such as AD [25].

### Co-expressed PPI modules are enriched in GenAge human aging genes

To validate the candidate modules and nodes, we determined whether GenAge, a database providing a comprehensive overview of aging-related genes in humans and model systems, contained these aging-related genes. Human aging genes were enriched within module 1 (Odds ratio (OR) = 11.3, 95% Confidence Interval (CI): 4.73-27.16, p < 0.001) and module 16 (OR = 13.1, 95% CI: 2.35-73.53, p = 0.019) (Table S6). We also found that human aging genes are enriched within the big subnetwork (OR = 4.57, 95% CI 2.38-8.76, p < 0.001) (Table S5). Notably, the genes contained in GenAge had higher centralities among all 1260 genes, and almost half had at least 10 degrees of centrality. The higher centralities indicate that the function of these genes may be more variable and essential in aging.

### Verification of functional roles using an established aged mouse model

**Figure 6:**
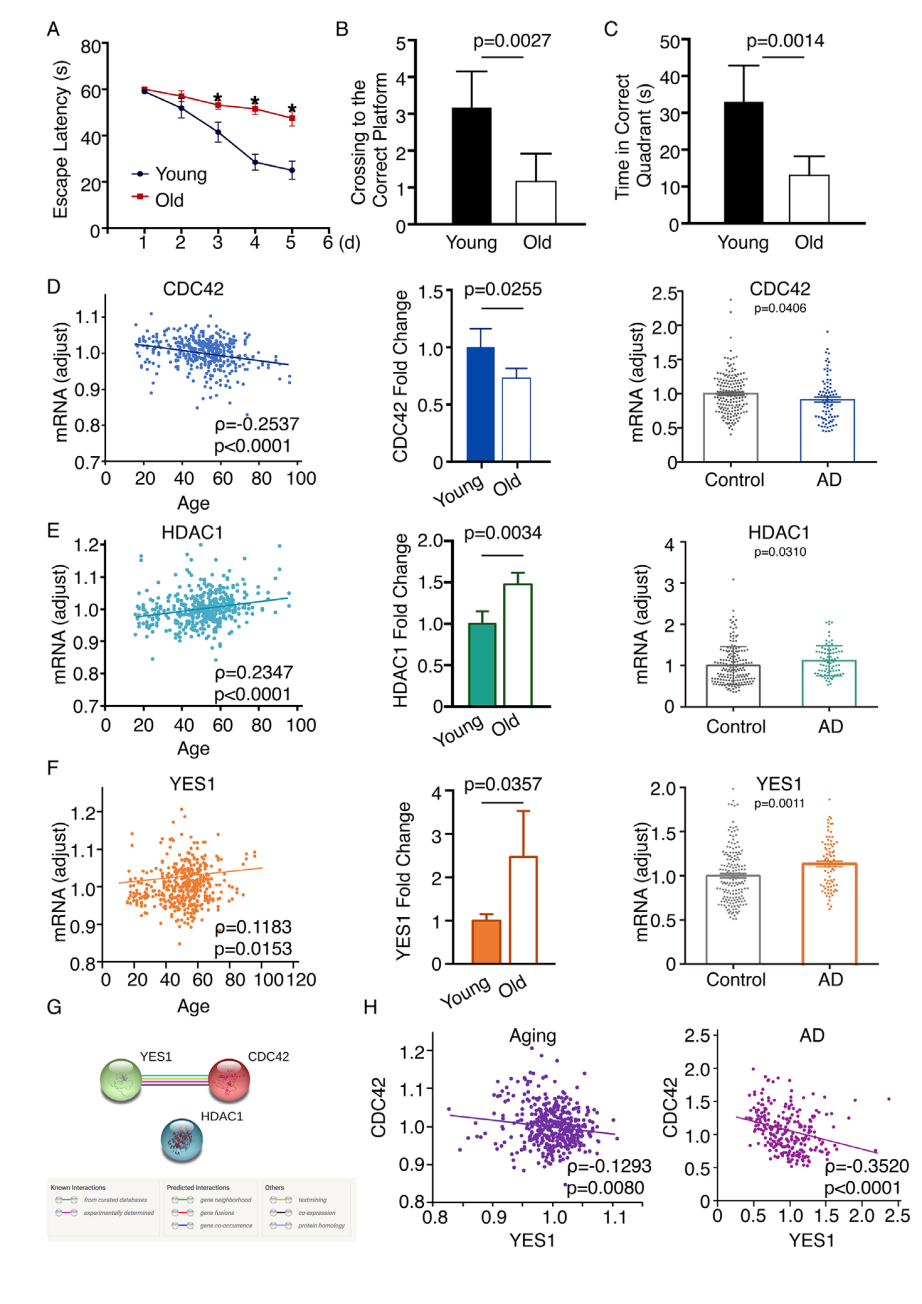
**Changes in CDC42, HDAC1 and YES1 mRNA expression in the frontal cortex of an aging mouse model.** (**A**) Escape latency on each day revealed a significant difference between younger and older mice on day 3 (p = 0.0313), day 4 (p = 0.0003), and day 5 (p = 0.0015) (n = 6). The average search errors of the younger and older mice did not differ in the first training trial (p = 0.3409) (n = 6). (**B**) (**C**) The numbers of times mice crossed to the correct platform (p = 0.0027) and the times in the correct quadrant (p = 0.0014) differed on the fifth day (n = 6). (**D**) Expression of CDC42 mRNA was associated with age through screening the GSE71620 dataset (ρ = -0.2537; P < 0.0001). Levels of Cdc42 mRNA were lower in older than younger mice (n = 6, p = 0.0255). Analysis of the GSE48350 dataset revealed levels of CDC42 mRNA are lower in the frontal cortex of people with AD than healthy subjects (p = 0.0406). (**E**) HDAC1 mRNA expression was associated with age through screening the GSE71620 dataset (ρ = 0.2347, p < 0.0001). Levels of Hdac1 mRNA were higher in older than younger mice (n=6, p = 0.0034). Analyzing the GSE48350 dataset revealed levels of HDAC1 mRNA are higher in the frontal cortices of people with AD than in those of healthy subjects (p = 0.0310). (**F**) Expression of YES1 mRNA was associated with age through screening the GSE71620 dataset (ρ = 0.1183; p = 0.0153). Levels of Yes1 mRNA were higher in older than younger mice (n = 6, p = 0.0357). Analyzing the GSE48350 dataset revealed levels of YES1 mRNA were higher in the frontal cortices of people with AD than in those of healthy subjects (p = 0.0011). (**G**) Functional link between YES1 and CDC42 determined using the STRING database to detect protein interactions. (H) YES1 mRNA levels associate with CDC42 in both neural aging and AD, as indicated by screening the GSE71620 (ρ = -0.1293; p = 0.0080) and GSE48350 (ρ = -0.3520; p < 0.0001) datasets.

To further verify the candidate genes screened with this network-based approach, we employed an established aged mouse model. Wild-type C57/BL6 mice maintained in a specific pathogen free (SPF) environment were used. Six-month-old mice comprised the young mature group, while 20-month-old mice comprised the old group. The Morris water maze was employed to evaluate the spatial learning and memory of two groups, which were assessed for 5 days. The escape latency significantly differed between the young and old mice (Day 3, p = 0.0313; Day 4, p = 0.0003; Day 5, p = 0.0015; Figure 6A), as did the numbers of crossings to the correct platform (Figure 6B) and the times in the correct quadrant on the fifth day (Figure 6C). qPCR performed to examine the mRNA expression in this model revealed that levels of Hdac1 and Yes1 mRNA were increased, while Cdc42 mRNA was decreased in the frontal cortex of the old group as compared to the young group, which was consistent with the results from the network analysis. By screening all age information in the GSE71620 dataset, we found that HDAC1, CDC42 and YES1 are associated with age. Moreover, by analyzing the GSE48350 dataset we found that HDAC1 and YES1 mRNAs were upregulated and CDC42 mRNA was downregulated in the frontal cortex of AD patients as compared to healthy subjects (Figure 6D-F). We then tested whether the three genes have an intrinsic relationship in aging or AD. By analyzing the STRING database, we found that there is only one significant connection between YES1 and CDC42 (Figure 6G). In both neural aging and AD (GSE71620 and GSE48350 datasets), CDC42 negatively correlates with YES1 (Figure 6F). We also verified 30 other genes using an established aging mouse model, and 18 were consistent with the results from the network analysis (Figure S2).

## DISCUSSION

Exploration of the age-associated genetic changes that occur in the human frontal cortex is a powerful approach for detection of the key mediators of the decline in brain function during both aging and neurodegenerative disease. In the present study, we built a transcriptional compendium of the genes involved in human frontal cortical function from five microarray studies that covered 161 younger and 155 older individuals. Ultimately, 1260 differentially expressed genes were identified and found to be enriched for several known terms of KEGG pathways related to age-associated neurological dysfunction. A PPI network-based analysis was used to further evaluate the relationship between these genes. That analysis suggests these genes interrelate with five significant subnetworks and 18 modules. We detected 33 hub genes that have at least 10 node degrees and occupy the top 10 percent of total genes. Among them, *HDAC1* was the centermost upregulated gene, followed by *YES1*. *CDC42* was the most significantly downregulated gene. We verified age-associated changes in these three genes within the frontal cortices of younger and older mice. These data demonstrate that Hdac1 and Yes1 are significantly increased in an established aging model, while Cdc42 is decreased. Analysis of GSE71620, which includes all ages showed that expression of CDC42, HDAC1 and YES1 mRNAs is affected by age. Moreover, analysis of the GSE48350 dataset showed the changes in the expression of those genes in AD are consistent with neural aging.

By catalyzing removal of acetyl groups from lysine residues in histones, HDACs are key determinants of chromatin structure. As mentioned, *HDAC1* is the centermost upregulated gene in our network analysis. Expression of HDAC1 was higher in aged mice than their younger counterparts. The enhanced expression of HDAC1 in the frontal cortex may be partly responsible for the observed age-related decline in neural function. In tauopathy and Huntington’s disease, HDAC1 is selectively increased in vulnerable brain regions such as the cortex and hippocampus, which contributes to the neurodegeneration in those ailments [26]. Inhibition of HDACs reverses cognitive deficits in AD [27] and aged mice [28]. Notably, levels of HDAC1 detected in blood paralleled the changes in the prefrontal cortex and hippocampus [29]. Assaying blood HDAC1 levels may thus be a useful method for screening patients at higher risk in cognitive decline. Our finding that HDAC1 is tightly linked to frontal cortex aging is consistent with an earlier report [26], which suggests our analysis method is reliable and practical.

*CDC42* was the most prominently downregulated gene across the five microarray datasets, and its downregulation was further confirmed in the aged mouse model. CDC42 is a master regulator of cell polarity. Activation of CDC42 correlates with loss of polarity, which may explain why CDC42 can accelerate the replicative senescence process in yeast cells and hematopoietic stem cells [30,31]. Based on results obtained using a transgenic model mouse ectopically expressing Cdc42-GTP (Cdc42GAP^-/-^ mice), it has been suggested that CDC42 promotes multiple aging-like phenotypes and shortens lifespan. MEF (mouse embryonic fibroblasts) cells from Cdc42GAP-/- mice exhibit lower population doubling potential, impaired DNA damage repair activity, accumulation of genomic abnormalities, and induction of senescent markers like p53, p16, p21, and senescence-associated β-gal (SA-β-gal) [32]. Whether or not Cdc42GAP^-/-^ mice experience neurologic dysfunction remains unknown, however, though it is known that CDC42 is crucial for learning and memory. Conditional knockout of Cdc42 in the postnatal forebrain contributes to a decline of synaptic plasticity and remote memory recall [33]. Levels of CDC42 expression differ in different brain regions. Within the cortex, Cdc42 expression greatly increases with aging, whereas it decreases in the hippocampus [34]. The function of CDC42 also depends on post-translational modifications, such as GTP binding. In addition, Cdc42 has two splice variants. The canonical prenylated isoform (Cdc42-prenyl) is the dominant splice variant and is widely expressed in various cell lines and the cerebellum. By comparison, the brain-specific isoform (Cdc42-palm) is the splice variant in the hippocampus and is mainly involved in the formation of dendritic filopodia and spines [35]. Considering the importance of the hippocampus and prefrontal cortex in memory and emotion, we suggest that changes CDC42, especially its brain-specific isoform, in the hippocampus and prefrontal cortex may be consistent with aging. CDC42 mRNA is downregulated in the dorsolateral prefrontal cortex of schizophrenia patients. This reduced expression of CDC42 may contribute to the decreased density of dendritic spines and cognitive dysfunction observed in schizophrenia [36–38]. At present, however, there is little data on the function of CDC42 in the frontal cortex during aging. CDC42 may be the key element in the interplay between the hippocampus and prefrontal cortex that governs memory and emotion.

*YES1* was the second most prominently upregulated gene found in our study. YES1 is one of the Src family kinases (SFKs), which have been implicated in the regulation of cell proliferation and differentiation during the development of the mammalian brain. After cerebral maturity, SFKs regulate neuronal plasticity and behavior through tyrosine phosphorylation of key substrates such as neurotransmitter receptors [39]. Fyn and Src, two other SFKs, contribute to the pathogenesis of AD by phosphorylating tau [40]. Saracatinib (AZD0530), a small-molecule inhibitor with high potency for Src and Fyn, was planned for a phase IIa multisite study for AD therapy [41]. By contrast, the function of YES1 in brain remains unknown. Current reports on YES1 nearly all focus on its role in promoting cell proliferation and inhibiting apoptosis in cancer. Interestingly, YES1 is essential for tyrosine phosphorylation of OCT2, which is distributed in cholinergic and monoaminergic terminals in the forebrain regions, and inhibition of YES1 diminishes OCT2 activity in central nervous system neurons *in vivo* [42,43]. In our study, YES1 was negatively related to CDC42 in both neural aging and AD. This suggests YES1 interacts with CDC42, which then function as a complex to regulate frontal cortex aging and age-related brain diseases.

The volume of available biological data has experienced explosive growth with the development of high-throughput technologies. Consequently, it is now important to integrate existing data so as to identify potential new information that can not only increase sample size but also enable merging of participants from different regions. Differential gene expression analysis usually focuses on a specific gene, which means the connection between genes can be overlooked. Because the integrative PPI network-based approach entails integration of data, it has several advantages. First, it reduces noise and increases power. Multiple PPI databases are literature-curated through the use of state-of-the-art quality control and validation. Second, biological processes involved in gene and protein expression are not isolated events. Network methods relate genes to each other and provide an essential organizing framework that places each gene within the context of its molecular system. Third, genes characterized by higher node degrees and betweenness centrality may function as hub genes. They may not only have a central role in a particular cellular function, they may also connect cellular components and regulate multiple tissues and systems [44]. In addition, we used a normal aging mouse model to verify the genes detected in our network analysis.

Nonetheless, the present study has several limitations that need to be addressed. First, our study lacks validation with an AD animal model. Second, although the selected genes have been evaluated in an established aging model, verification using human samples with a detailed medical history would enhance the reliability of our results. Finally, a specific knock-in/out mouse model will provide better understanding for the underlying mechanisms involving CDC42, YES1 and HDAC1 in the functional decline during aging and AD.

In summary, by applying a network-based approach, we identified *HDAC1* and *CDC42* as key mediators of age-related changes in neural function, which is in accordance with earlier reports. In addition, we identified a novel gene, *YES1*, which is potentially critical to frontal cortex aging and is thus a potential biomarker and therapeutic target underlying brain aging. The expression patterns of *CDC42*, *HDAC1* and *YES1* during neural aging are consistent with AD, and may establish a new molecular connection between aging and AD. These findings thus suggest that network analysis provides a framework to screen for potential biomarkers underlying brain aging, and will serve as a novel input to improve our understanding of the aging process.

## MATERIALS AND METHODS

### Building the frontal cortex gene expression compendium

Gene expression data from microarray studies were downloaded from the Gene Expression Omnibus (GEO) using the terms (Frontal Lobes) OR (Frontal Cortex) OR (Anterior Central Gyrus) OR (Superior Frontal Gyrus) OR (Prefrontal Cortex) AND (aging) AND (transcriptional profiling). Microarray studies using RNA samples from human frontal cortices were included in our study. The basic characteristics used to identify the studies included first author, year of publication, dataset, platform, region, sample number and type. Because it has been shown that the changes in gene expression across cortical regions occur mainly when people are in their 20’s and 60’s [17,18], statistical group comparisons were made between subjects classified as younger (20-40 years) or older (60-90 years). Only microarrays that contained three or more samples in both the younger and older group were included in our study. Using these criteria, five studies were ultimately selected, all of which exhibited a common transcriptional signature in the prefrontal cortex throughout the aging. Postmortem human brain materials were obtained from subjects without a medical history of neuropathology, drug use, alcohol abuse, or psychiatric illness.

### Microarray meta-analysis

We conducted a microarray meta-analysis using INMEX (a web-based tool for integrative meta-analysis of expression data) [45]. INMEX is in accordance with the Preferred Reporting Items for Systematic Reviews and Meta-Analyses guidelines for meta-analysis [46]. All gene probes were converted to a common Entrez ID using the gene/probe conversion tool in INMEX. After changing to Entrez ID, all datasets were preprocessed through a log2 transformation and Variance Stabilizing Normalization (VSN), followed by quantile normalization. Each individual dataset was visualized in box plots to ensure identical distribution among the samples. Differential expression analysis was performed independently for each dataset using INMEX with an FDR of 0.05 and a significance of p < 0.05. The moderated t test was based on the Limma algorithm. In INMEX, the results from individual microarray datasets are only for reference comparison and are not required for the subsequent steps of the meta-analysis [45]. For the meta-analysis, we used the Fisher’s method, a fixed effect model, and Vote counting (a significance level of p < 0.001, p < 0.001, votes number < 2). Fisher’s method (-2*∑Log (p)) is a statistical approach widely used in meta-analysis for combining P values from different studies independently of the sample size. This method is generally more sensitive than a combined analysis (i.e. it detects more DE genes). Fixed effect models combine effect sizes, and the estimated effect size in each study is assumed to come from an underlying true effect size plus measurement error. A fixed effect model can be selected based on statistical heterogeneity estimated using Cochran’s Q tests. Estimated Q values that approximate a chi-squared distribution suggest the fixed effect model assumption is appropriate. This method is usually conservative (fewer DE genes are detected, but with higher confidence). Vote counting is a meta-analysis method in which differentially expressed genes are first selected based on a threshold (a significance level of p < 0.05) to obtain a list of DE genes for each study. The vote for each gene can then be calculated as the total number of times it occurs in all DE lists. The final DE genes can be selected based on a minimal number of votes set by the user.

### Pathway enrichment analysis

The pathways of the identified proteins were classified using the DAVID program (http://david.abcc.ncifcrf.gov) for KEGG annotation. To determine GO categories, we used Cytoscape - BinGO. A custom annotation file was created using the built-in annotation file for GO biological processes.

### Network-based meta-analysis and extracting co-expressed PPI modules

A network-based meta-analysis was performed using NetworkAnalyst and STRING (Search Tool for the Retrieval of Interacting Genes/Proteins). Microarray datasets were processed as described above. Network construction was restricted to contain only the original seed proteins. Protein-protein interactions were predicted using the STRING database v10.5 (http://www.string-db.org/). Proteins that linked to each other were detected based on experimental determination, curated databases, gene neighborhood, gene fusions, gene co-occurrence, co-expression, text mining and protein homology. An extended network was constructed using a minimum required interaction score (> 0.9) as the selection parameter, which implies that only interactions with a high level of confidence were extracted from the database and considered as valid links for PPI networks.

### Morris water maze

Wild-type C57/BL6 mice were purchased from the Experimental Animal Center at Tongji Hospital (Wuhan, China). All animals were housed at the animal care facility at Tongji hospital. These animal studies were approved by the Institutional Animal Research Committee of Tongji Hospital. The experimental protocols were approved by the Institutional Animal Care and Use Committee (Approved number: TJ-A20160503). Six-month-old mice comprised the younger group, while 20-month-old mice comprised the older group. The maze consisted of a circular tank (diameter, 150 cm) filled with water (27 °C) to which powdered milk was added. An escape platform (diameter, 10 cm) was located 1 cm beneath the water surface. Acclimation to the water maze was performed on day 0, after which learning trials were conducted on days 1-5. The open field test was performed on day 5. Mice participated in 3 trials per day for 5 consecutive days using a 60-s inter-trial interval.

### Quantitative real-time polymerase chain reaction

The frontal cortex was collected from younger and older mice. Total RNA was extracted using a HI Pure RNA extract kit (Magen, China) and converted to cDNA using a ReverTra Ace qPCR RT kit (TOYOBO, Japan). Relative mRNA expression was detected using RT-PCR performed on an ABI Step One Plus (Applied Biosystems, USA) with SYBR green PCR master mix (TOYOBO, Japan). The primer sequences are as follows: GAPDH — forward, 5′ - AATGGTGAAGG TCGGTGT - 3′; reverse, 5′- GTGGAGTCATACTG GAACATGTAG - 3′; CDC42 — forward, 5′ - TGCT CTGCCCTCACACAGAAAG - 3′; reverse: 5′ - GCGG CTCTTCTTCGGTTCTG - 3′; and HDAC1 — forward, 5′ - CTCACCGAATCCGCATGACT - 3′; reverse, 5′ - GGCTTTGTGAGGACGGTAGA - 3′, YES1 — forward, 5′ - AATGAGGACCAGA GGGTAGGG - 3′; reverse, 5′- CATTATCAAATCCGCTCGCTCC - 3′. The sequences of other primers used in this study are shown in Table S7. The fold change in relative mRNA expression was calculated using the 2^-ΔΔCt^ method.

### Statistical analysis

Network-based microarray meta-analysis was performed using INMEX and NetworkAnalyst. In the Morris water maze experiment, measures of performance during acquisition trials (i.e., escape latency) were averaged within each day for each animal. Data are reported as the mean ± standard error. To evaluate differences among the days, data were analyzed using two-way repeated measures ANOVA, with day as the within-subjects’ factor and treatment as the between-subjects’ factor. Differences between the two groups were analyzed using one-way ANOVA, after which Fisher’s LSD was used for post hoc comparisons. For RT-PCR experiments and the analysis of the GSE48350 dataset, statistical significance was determined using Student’s t-test (for independent or dependent samples, as appropriate). Values of p < 0.05 (two-tailed) were considered significant. Spearman correlation analysis was used to determine statistical significance for CDC42, HDAC1 and YES1 adjusting for age in the GSE71620 dataset. Other statistical analyses were performed using SPSS 19 software (SPSS Inc., USA).

## SUPPLEMENTARY MATERIAL

Table S1

Table S2

Table S3

Table S4

Table S5

Table S6

STable S7

Figure S1

Figure S2

Data S1
