# Supplementary figures and images for: Re-exploring the core genes and modules in the human frontal cortex during chronological aging: insights from network-based analysis of transcriptomic studies
Source: Aging (Albany NY). 2018 Oct 20;10(10):2816–31. doi: 10.18632/aging.101589 (PMC6224233; doi:10.18632/aging.101589)

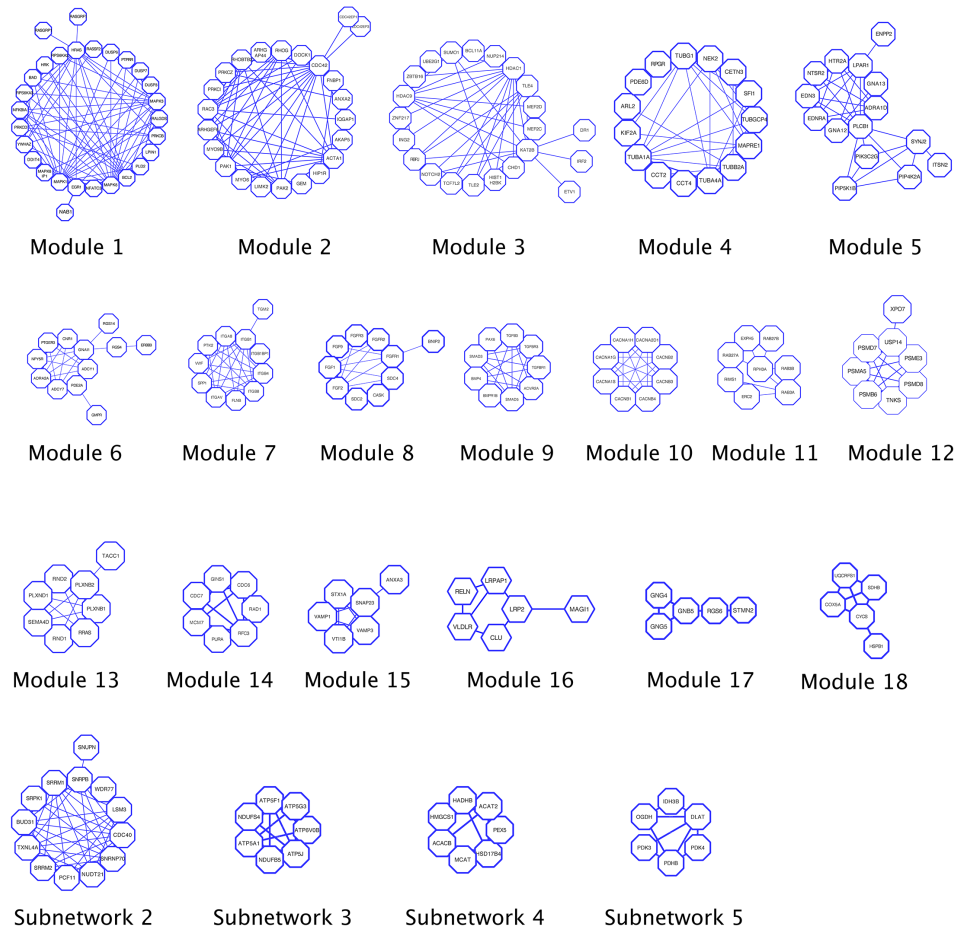

**Figure S1. Overview of consistently detected coexpressed PPI modules.**

Supplement: Figure S1 [file aging-10-101589-s008.pdf]

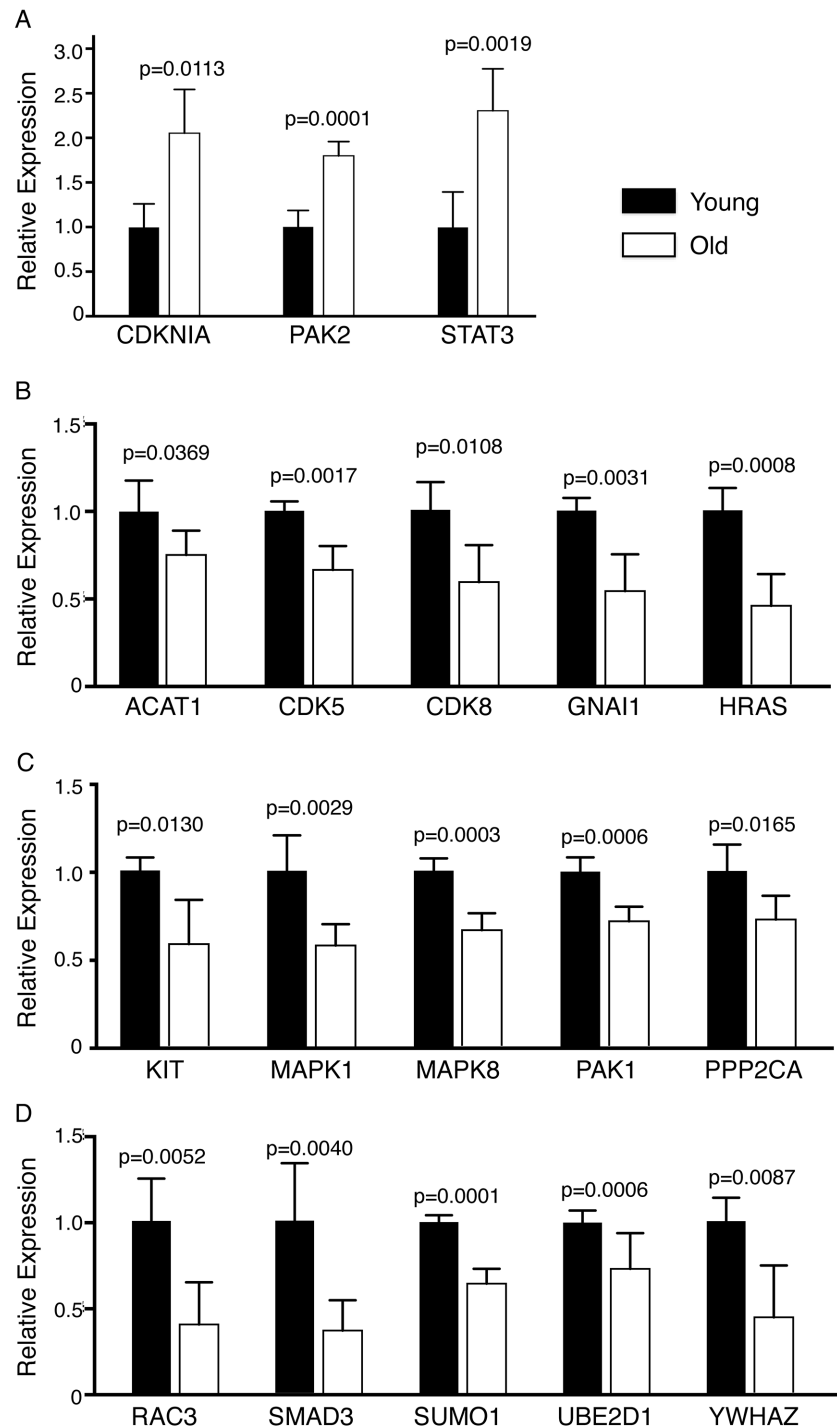

**Figure S2. Eighteen genes consistently detected in the results from the network analysis.**

Supplement: Figure S2 [file aging-10-101589-s009.pdf]
